# Influence of prey availability on habitat selection during the non-breeding period in a resident bird of prey

**DOI:** 10.1186/s40462-023-00376-3

**Published:** 2023-03-07

**Authors:** Roman Bühler, Kim Schalcher, Robin Séchaud, Stephanie Michler, Nadine Apolloni, Alexandre Roulin, Bettina Almasi

**Affiliations:** 1 Swiss Ornithological Institute, Seerose 1, 6204 Sempach, Switzerland; 2 Department of Ecology and Evolution, University of Lausanne, Building Biophore, 1015 Lausanne, Switzerland; 3 Agroecology and Environment, Agroscope, Reckenholzstrasse 191, 8046 Zurich, Switzerland

**Keywords:** Full annual cycle, Non-breeding period, GPS, Small mammals, Resource selection, *Tyto alba*, Bird of prey, Habitat selection, Biodiversity promotion areas

## Abstract

**Background:**

For resident birds of prey in the temperate zone, the cold non-breeding period can have strong impacts on survival and reproduction with implications for population dynamics. Therefore, the non-breeding period should receive the same attention as other parts of the annual life cycle. Birds of prey in intensively managed agricultural areas are repeatedly confronted with unpredictable, rapid changes in their habitat due to agricultural practices such as mowing, harvesting, and ploughing. Such a dynamic landscape likely affects prey distribution and availability and may even result in changes in habitat selection of the predator throughout the annual cycle.

**Methods:**

In the present study, we (1) quantified barn owl prey availability in different habitats across the annual cycle, (2) quantified the size and location of barn owl breeding and non-breeding home ranges using GPS-data, (3) assessed habitat selection in relation to prey availability during the non-breeding period, and (4) discussed differences in habitat selection during the non-breeding period to habitat selection during the breeding period.

**Results:**

The patchier prey distribution during the non-breeding period compared to the breeding period led to habitat selection towards grassland during the non-breeding period. The size of barn owl home ranges during breeding and non-breeding were similar, but there was a small shift in home range location which was more pronounced in females than males. The changes in prey availability led to a mainly grassland-oriented habitat selection during the non-breeding period. Further, our results showed the importance of biodiversity promotion areas and undisturbed field margins within the intensively managed agricultural landscape.

**Conclusions:**

We showed that different prey availability in habitat categories can lead to changes in habitat preference between the breeding and the non-breeding period. Given these results we show how important it is to maintain and enhance structural diversity in intensive agricultural landscapes, to effectively protect birds of prey specialised on small mammals.

**Supplementary Information:**

The online version contains supplementary material available at 10.1186/s40462-023-00376-3.

## Introduction

Annual cycles organize living beings in different periods (e.g., dispersal, migration, breeding, non-breeding) which can vary in duration and geographical location depending on the species [1]. In temperate regions, periods are generally accompanied by meteorological changes, where lower temperatures in autumn and winter lead to changes in productivity on several trophic levels, affecting primary producers and their consumers [2]. Species respond to changes in resource availability with different strategies. While migratory species leave breeding habitats when resources decline and move to areas with higher resource availability, resident animals react to changes in resources by shifting their hunting grounds and/or exploiting alternative resources [3, 4]. Such seasonal changes do not only affect individual performance in their current state but can also carry over and affect future events, such as reproduction [5]. Considering the important influences of the non-breeding period on individual performance and survival and the resulting effects on population development, this demanding period should receive more attention, especially in the light of species conservation.

Birds react sensitively to food shortage, making food availability one of the most important factors to address in conservation research [6–10]. Local changes in food availability potentially have a high impact on sedentary animals with high prey specialisation, as they are limited in flexibility regarding resource exploitation. For birds of prey specialised on small mammals, resource availability fluctuates as prey densities may show multi annual cycles, as well as density changes within an annual cycle [7, 11–13].

Within the annual cycle, fluctuations in prey densities in a habitat can be highly dependent on habitat characteristics. Small mammals living in semi-natural habitats, such as agricultural dominated landscapes, are often exposed to sudden periodic disruptions. These disruptions affect local population densities within a short period of time and have the potential to influence future densities on a large scale [14, 15]. An example of such a disruption is the harvesting of intensively managed crop plantations: while small mammal abundance within seasonal crop may be high in summer, harvesting leads to sudden population changes within these areas [14–18]. The main drivers of such population changes after harvest are either direct mortality due to mechanical treatment, increased predation due to reduced vegetation cover, or a shift of activity in less disturbed and more suitable habitats [14, 18–20]. Biodiversity promotion areas (e.g., wildflower strips, rotational fallows), semi natural habitats (hedges) and field margins as well as perennial crops represent such less disturbed habitats [18, 21–26]. These habitats can act as refuge during periods of disturbance and can allow small mammals to reuse or recolonise intensively managed agricultural fields after disturbance [23–25, 27]. The resulting seasonal change in spatial distribution of prey is likely able to influence habitat selection of their predators. Thus, we hypothesize that the preference for different habitat categories should be plastic and shift according to the availability of resources within those habitats at the corresponding time.

As a resident bird of prey, the barn owl (*Tyto alba*) is sensitive to reduced food availability [28] and harsh winter conditions [29]. At low ambient temperatures, the metabolic rate increases, resulting in higher energy requirements to cover baseline energetic demand [30, 31]. At the same time prey availability can be drastically reduced due to low abundance and activity of small mammals [7, 11, 12]. Additionally, harsh winters with a closed snow cover reduce accessibility of prey [29]. If such unfavourable circumstances persist for several days (starvation limit ~ 7 days, [31]) this not only affects the survival of individuals but can also lead to the extinction of regional populations [29].

With agricultural intensification the European barn owl suffered from a strong decline across Europe and is listed as vulnerable in Switzerland [32]. The availability of nesting sites and foraging habitats are the most important factors affecting population size [33, 34]. The installation of nest boxes can increase breeding success, but probably only has little effect on the survival outside the breeding period. The habitat requirements of barn owls, especially during the non-breeding period, are still not well studied. We need a better understanding of the habitats in which prey occur at sufficiently high densities during the non-breeding period and are also accessible to predators. Identifying these habitats is key to implementing further conservation measures to ensure the continued existence of resident birds of prey in a fast-changing landscape. In this study, we (1) quantified barn owl prey availability in different habitats throughout the annual cycle, (2) quantified the size and location of barn owl breeding and non-breeding home ranges, (3) assessed habitat selection in relation to prey availability during the non-breeding period, and (4) discussed the changes in habitat selection between the breeding and non-breeding period. We expect to see a seasonal shift in prey availability from intensively managed to less disturbed habitats and a corresponding shift in predator habitat selection towards these prey rich habitats.

## Material and methods

### Study area and study species

The study was carried out in western Switzerland (46°49′ N, 06°56′ E) in an area of ~ 1000 km^2^. The study area consists of four geographical regions (see Additional file 1: S1.1), two lowland regions dominated by intensive arable farming (plain of Orbe, plain of the Broye), and two hilly regions dominated by grazing and arable farming (Haut-Fribourg, Gros de Vaud). One inhabitant of such agriculturally dominated landscapes is the barn owl (*Tyto alba*), a medium-sized raptor which mainly preys on small mammals. The Swiss population almost exclusively breeds in nest boxes installed in or on buildings. More than 400 nest boxes for barn owls have been installed throughout the region since 1985. The breeding period extends from February to September [35], followed by a resident non-breeding period.

### Small mammal monitoring

While *Arvicola, Microtus, and Apodemus* species represent the staple prey of European barn owls in Central Europe, barn owls are also known to prey on other small mammals like *Myodes* and *Sorex/Crocidura* species [34, 36]. Voles (*Arvicola *sp., *Microtus *sp.*)* inhabit open habitats, especially grasslands, and are partly active below ground using rather small areas (e.g., common vole, *Microtus arvalis*: 200 m^2^) [15, 27, 37]. *Apodemus* species live in open habitats as well as wooded areas, are mainly active above ground, and can show large home ranges (e.g., wood mice, *Apodemus sylvaticus*: 4900–14,400 m^2^) [18]. We monitored potential prey for barn owls within our study region from 2015 to 2021 by using two indirect methods: First, we counted signs of recent vole activity (heaps, holes, and runways) along short transects (5 × 1 m [38]). Such signs are unique and well distinguishable from naturally occurring variation in vegetation [39]. To ensure that the signs were from recent activity, we looked for fresh droppings and remains of recently cut vegetation. Counting such signs of activity gives us information about the presence and activity density (combination of activity and abundance) of voles. Second, to assess the availability of all small mammal species, we laid plates covered with a thin layer of graphite along the transects (method adapted from [40]). As small mammals walk across these track-plates they leave distinct traces on the thin graphite layer. The number of these traces provides information about presence and the activity density of small mammals. Combining both methods, information for the presence and activity density of both voles and total small mammals are obtained.

In each of the four geographical regions in our study area, we defined four plots of 9 km^2^ (see Additional file 1: S1.1) where small mammal activity density was surveyed in five different habitat categories (crop rotation, grasslands, border structures, forest, biodiversity structures) every second month (six sessions per year). Nine transects were walked in each habitat category per plot and, region and recent signs of vole signs (heaps, hole, runways) were counted. In addition, one track-plate covered with graphite was laid out along each transect during three consecutive nights. Small mammal traces were subsequently counted on each track-plate. This resulted in a total of 720 transects and an equal number of track-plates for each 2-months session (see Additional file 1: S1.2).

Track-plates and transect counts correlate well with estimates of relative abundance from live trapping [40, 41], are less time-consuming than live traps, and are therefore best suited to be used for large-scale small mammal monitoring. Track-plate and transect counts reflect the activity density of potential prey and are likely to correlate with prey availability, as areas with more active [42] and abundant prey are generally preferred by predators. As vole populations in central Europe vary synchronously over large spatial scales [43], we are confident that the spatial resolution of our small mammal monitoring design is appropriate to estimate prey activity density over the scale of our geographical regions.

### GPS deployment and home range

To study habitat use during the non-breeding period, we equipped adult barn owls with GPS devices. We captured the owls during the breeding period when they were feeding the nestlings, by placing a sliding door at the nest box entrance, which the owl triggers when it enters [44]. After capturing, the individual was measured, and its sex determined based on the presence or absence of a brood patch. The GPS-tag (Gipsy-5 and Axy-Trek, Technosmart, Italy) was combined with a VHF-transmitter (µTag, Swiss Ornithological Institute) and attached as a wing loop harness with spectra tubular tape (4.7 mm, polyethylene, Bally Ribbon Mills, US). The combined tag weighted < 13 g (which is < 5% of body mass, mean body mass of birds before tagging: 297 g, range: 260–440 g, limit for equipment: > 260 g). We used two different GPS modules (Gipsy-5 and Axy-Trek) which both must be retrieved to read the data. GPS tags were programmed to start with a delay to capture locations as far into non-breeding period (October to February) as possible. Once activated, one position was recorded every hour for two halfnights a week (7–12 p.m. and 0–4 a.m. UTC), resulting in an average of 6.6 locations per week. The VHF tag was programmed to be active for 1 week the following spring. During this week, we searched for the tagged individuals, checked the status of the birds and recovered the GPS tags of dead owls. Individuals breeding in the year after GPS deployment were recaptured during chick feeding as described above. Ten days before GPS deployment for the non-breeding period, the owls were captured a first time to be equipped with a GPS tag to record breeding habitat use for another study [44, 45]. For this study purpose GPS tags were programmed to recorded with a resolution of 1 location every 60 s for the following 10 days. After these 10 days, the owls were re-captured and equipped with the GPS for the non-breeding period. The breeding period data are used in this study to compare size of breeding and non-breeding home ranges, their overlap and distances from the breeding nest box to the centroid of the breeding and non-breeding home ranges. All GPS data have been uploaded to Movebank (non-breeding period ID: 1433219445; recovery rate of GPS tags in 2018: 55%, females: 22 out of 37, males: 20 out of 39; 2019: 68%, females: 22 out of 41, males: 37 out of 46, and breeding period ID: 231741797).

Throughout the non-breeding period (September–February), we obtained 10,142 GPS locations from 65 different individuals (males: 32, females: 33) from 74 (out of 101) deployments (duration of recording: 31–180 days, mean: 132 days). GPS data were filtered for duplicated locations and aberrant positions (528 points removed). The latest GPS modules (Axy) included triaxial accelerometers, resulting in slightly higher energy consumption compared to the GPS-only modules (Gipsy). To compensate for this difference, the Axy modules were programmed to skip fixing locations during periods of bird inactivity. To obtain comparable data, we resampled the Gipsy data by removing locations which were close in time and space, i.e., where the bird appeared to be inactive (birds that did not move more than 30 m within 120 min). The resampling process removed 1195 locations, and we ended up with a dataset of 8419 locations.

Non-breeding and breeding home range sizes were calculated using a minimum convex polygon (MCP), containing 95% of locations for each animal. We used the function hrBootstrap [46] to assess the saturation of our estimated home ranges. We considered home ranges to be saturated if the increase in area between the last 3 iterations of the stepwise increase did not exceed 15%. We were able to estimate stable non-breeding home ranges for 51 (males: 27, females: 24) out of 74 deployments. Home ranges were estimated from 30 up to 212 locations per individual with a mean of 130 locations (females: mean: 141, range: 38–194, males: mean: 119, range: 30–212). For 46 (males: 24, females: 22) of the 51 non-breeding home ranges, data were available to calculate breeding home range of the previous reproductive period. Breeding home range was estimated based on 370 up to 5001 locations per individual with a mean of 3086 locations (females: mean: 3185, range: 1017–4516, males: mean: 2995, range: 370–5001).

### Habitat variables and home range composition

Information on the coverage of forest, urban area, hedges, single trees, roads (field path, minor, and major roads, highways, railways) and border structures (1 m buffer around roads) was obtained from the TLM3D database of the Swiss topographic institute (TLM3D, resolution: 0.2–3 m, Federal office of topography: Swisstopo, Seftigenstrasse 264, 3084 Wabern). The different categories were buffered to create the layers used in the final analysis (for details see Additional file 1: S1.3). Agricultural land was divided into four different habitat categories: intensive grassland (intensively managed pastures and meadows), extensive grassland (extensively managed pastures and meadows), crop rotation, wildflower strips and rotational fallows. Information on these habitat categories were extracted from the cantonal GIS-layer (canton Vaud: all agricultural fields, canton Fribourg: only permanent cultures) and supplemented with data from habitat mapping in the canton Fribourg (for details see Additional file 1: S1.4/1.5). Finally, hedges, single trees (source: TLM3D) as well as wildflower strips and rotational fallows (source: cantonal layers, part of biodiversity promoting areas) were summarized under the term biodiversity structures. To obtain habitat composition at the home range level during non-breeding period, coverage of the five main habitat categories (crop rotation, extensive grassland, intensive grassland, forest, and urban area) within each of the 51 previously calculated non-breeding MCP was extracted.

To model resource selection within non-breeding home ranges (third order selection [47]), we randomly selected available locations at a ratio of 1 used versus 100 available locations within each home range (95% MCP). With the 95% MCP boundaries used for sampling of available locations, only used locations within the 95% MCP were considered for the analysis (7534 locations used and 753,400 available locations). We then placed a 75 × 75 m square over each used and available location, hereafter referred to as focal area. This focal area was divided into a central 25 × 25 m (containing the GPS point) and eight surrounding 25 × 25 m grid cells. From each grid cell the proportion of area covered by the 5 habitat categories (crop rotation, intensive grassland, extensive grassland, urban area, forest) was extracted and the central grid cell (of the 3 × 3 cell grid) was weighted twice as much than the surrounding grid cells (0.2 vs 0.1). Additionally, the number of different habitat categories was extracted and used to describe structural richness. By considering a broader and weighted focal area rather than just the central grid cell, we minimized the risk of missing meaningful signals due to GPS localization errors (mean accuracy with settings used: 20 m). Focal areas for which less than 50% and more than 120% of their total area was described, were excluded from the analysis. This was the case whenever information describing the area was missing, or the same area was described by contradictory, overlaying layers (605 removed out of 7534 used and 85,303 removed out of 753,400 available locations). For an initial analysis of habitat selection within the home range, we reduced our dataset to one habitat category per focal area by assigning each used and available location the category with the highest coverage within the focal area (dominant habitat category). We removed all locations for which the dominant habitat category on the focal area was unknown (used: 42 removed out of 6929, available: 6968 removed out of 668,097).

### Statistical analysis

#### Vole and small mammal activity density

The numbers of vole signs (transect method, hereafter vole activity density) and the number of small mammal traces (track-plate method, hereafter small-mammal activity density) were analysed separately by fitting a generalized additive model (GAMM, [48]). While the total number of traces or signs, on the track-plates or along transects, served as response variable, region, observation round, habitat category, and temperature (for transects: mean from 10 days before transect counts, for track-plates: mean of the 3 days on which track-plates were exposed) were included as explanatory variables. Fitted values for a given time for each habitat structure in each region are estimated separately for transect and track-plate counts and are used as an index of vole and small mammal activity density (abundance, and activity). Model fit was evaluated using shinystan [49], which provides visual and numerical summaries of model parameters and convergence diagnostics for Markov Chain Monte Carlo (MCMC) simulations.

**Fig. 1:**
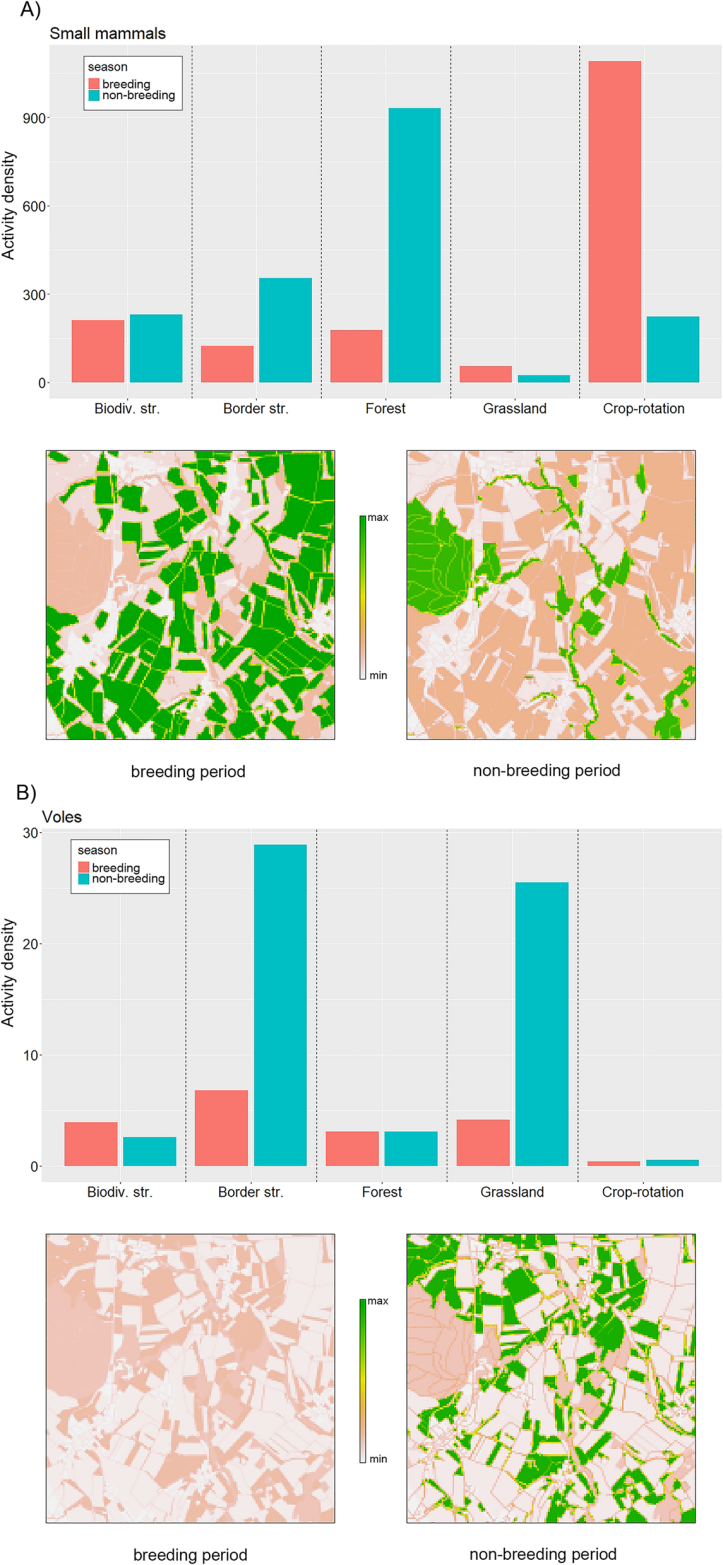
**A** Small mammal and **B** vole activity densities during breeding (May–June, red) and non-breeding period (November–December, blue) for biodiversity structures, border structures, forest, grassland and crop-rotation fields within the region Orbe for the year 2018. Lower graphs: visual representation of small mammal and vole activity density on the landscape level for owl breeding and non-breeding period (left: breeding period, right: non-breeding period). Colour scale indicates the activity density value for a certain area and is comparable within indices but not between

To illustrate potential differences between the breeding and the non-breeding period in vole and small mammal activity densities, we visualized the vole and small mammal activity density for the November–December sampling round (non-breeding period) and the May–June sampling round (breeding period) in the “Plain of Orbe” region in 2018. To do so, we multiplied the activity density by the area covered by each habitat category within a 25 m resolution grid (Fig. 1). Using the same procedure, we interpolated small mammal and vole activity density for our 75 m × 75 m focal areas in the analysis later on.

Because the two activity densities showed different patterns depending on habitat types and period, with different implications for barn owl availability and accessibility, they were interpreted and analysed separately in the following steps.

#### Home range size and location of home ranges

To assess if home range size differed between male and females and between periods, we ran linear models with log-transformed home range size as the response variable and period (breeding/non-breeding), sex and year as explanatory variables. Individual identity was included as random effect. To assess if the spatial distributions of locations of breeding and non-breeding home ranges differ, we measured (1) the distance from the centroid of the breeding home range to the breeding nest box, (2) the distance from the centroid of the non-breeding home range to the previous breeding nest box, (3) the distance from the centroid of the non-breeding home range to the future breeding nest box. We then built a linear model with log-transformed distance as response and the type of distance (1,2,3) as well as sex as explanatory variable. Individual and nest box ID were included as random effects. Posterior distribution was obtained by simulating 1000 values from the joint posterior distribution of model parameters using the “simulate_model” function of the “parameters” package [50]. The means of the simulated posterior distribution were used as estimates and the corresponding 2.5% and 97.5% quantiles as the upper and lower bounds of the 95% credible interval (CrI). Effects were interpreted as meaningful when they differed from 0 (95% CrI not containing 0).

#### Resource selection

To compare habitat variables at used (GPS fixes) versus available locations within the owls’ home range (third-order selection [47]), we used a resource selection function (RSF; [51]). The sampling design used to collect our data (1 fix/hour) should meet the assumption of independence. To generate a population level RSF function, we used a generalized linear mixed effects model with a weighted logistic regression, binomial error distribution and a logit-link function (glmmTMB; [52]).

To account for the non-independence of the data derived from the same individual we included a random intercept for each individual and deployment (hereafter tag deployment) in our models [53]. Following the recommendation of Muff and Fieberg [54] we included random slopes for our explanatory variables whenever possible to account for individual-specific variation in habitat selection.

**Fig. 2:**
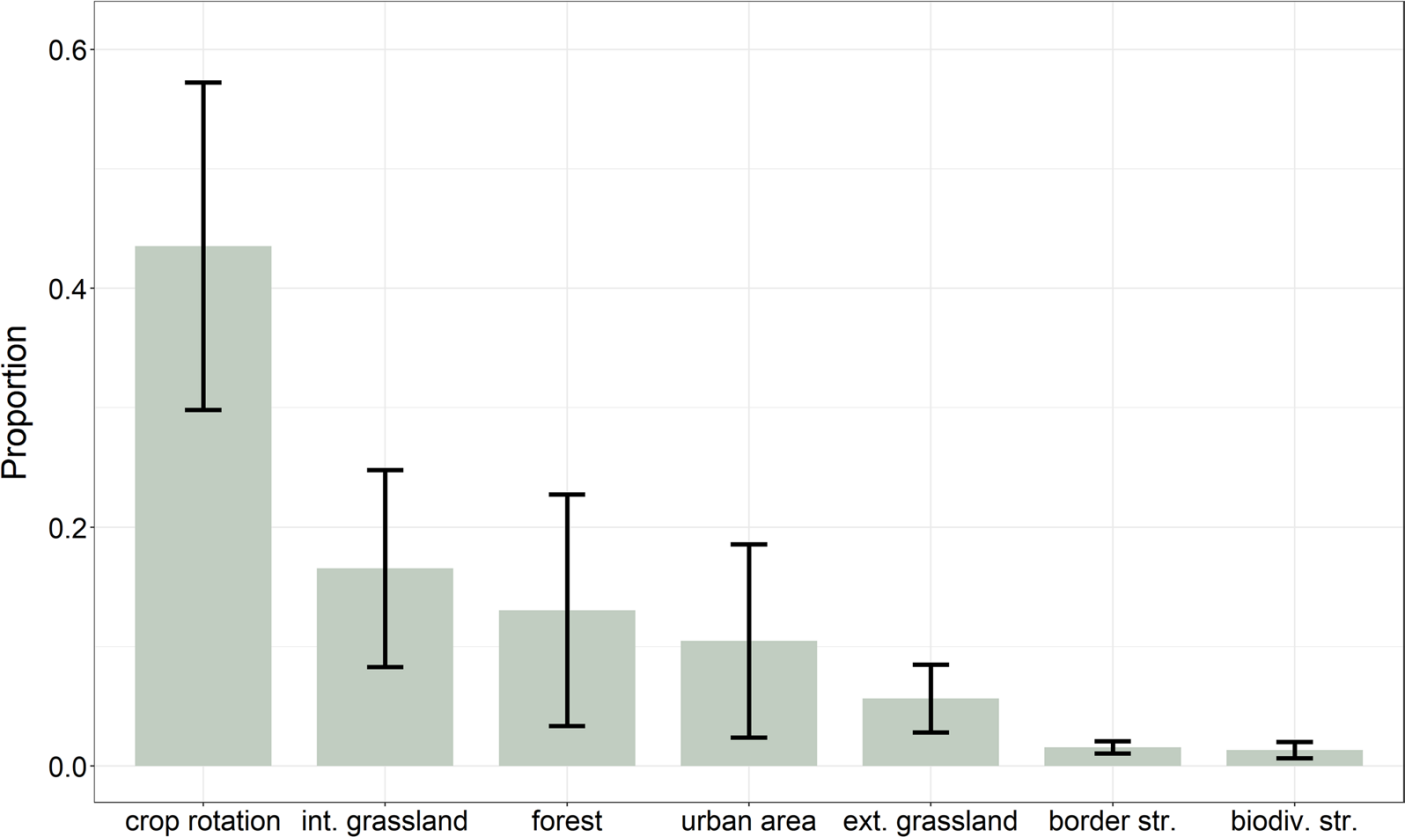
Visualisation of the proportion of habitat categories present within the 51 non-breeding home ranges, with the corresponding standard deviation

First, we built an RSF with 6887 used and 661,129 available focal areas (ratio: 1:96), as response variable in relation to the dominant habitat category of the focal area (intensive grassland, extensive grassland, crop rotation, urban area, forest), biodiversity structure (presence or absence of hedges, trees, wildflower strips, rotational fallows within the focal area), structural richness (number of different habitat categories within the focal area), and sex (male/female). To determine whether the presence or absence of biodiversity structures influenced habitat category choice, we included the interaction with the dominant habitat category in a second model. We included a random intercept for each individual and deployment (hereafter tag deployment) and random slopes for habitat category, biodiversity structure, and structural richness in the model. To meet the assumptions of an inhomogeneous point process model, we assigned a weight of 5000 to available locations and a weight of 1 to all used locations [55, 56]. In our sensitivity analysis, which we performed with ratios of 1:10, 1:50 and 1:100, estimates stabilized at a ratio of 1:10. For the final analysis, we chose the highest ratio of 1:100, because computation time was not limiting in our case. The exponential value from the β estimates is referred to as relative selection strength (RSS) [57]. RSS values from used-available studies do not represent true probabilities but are proportional to the probability of selection. We choose crop rotation as the reference level for habitat category because it is the most abundant habitat category within owl home ranges (Fig. 2).

In a second step, we wanted to test whether the activity density of small mammals or voles per focal area influences selection and whether the presence of biodiversity structures influences selection. To calculate vole or small mammal activity density per focal area, the area of the specific habitat category of the focal area (crop rotation, extensive grassland, intensive grassland, border structures, biodiversity structures) was multiplied by the corresponding vole or small mammal activity density (region and time) and summed up over the focal area. This gives us one value for vole and one for small mammal activity density per focal area. We then ran a second glmmTMB model with vole and small mammal activity density per focal area, the presence or absence of biodiversity structure and sex as explanatory variables. Tag deployment (unique identifier for each individual per year) was included as random intercept and vole activity density per focal area, small mammal activity density per focal area, and biodiversity structures were included as random slopes.

In a third step, we modelled whether focal area selection is dependent on the combined prey activity density within the different habitat categories (total prey activity density). To combine the activity density of voles and small mammals per habitat category and focal area, we first multiplied the area of each habitat category (crop rotation, grassland, border structures, biodiversity structures) within each focal area by the corresponding value for the activity density per habitat category and focal area for voles and small mammals separately. These activity densities per habitat category and focal area, after being centred and scaled, were summed to obtain a total activity density per habitat category and focal area. We then ran a model with total prey activity density per foraging habitat category and focal area, sex and structural richness as explanatory variables. Tag deployment was included as random intercept and prey activity density for each habitat and focal area as random slopes. To reduce model complexity and allow the model to converge the two types of grassland (extensive and intensive) were merged.

As the focus of the analyses of the second and third step described above was on prey availability in the foraging habitats of barn owls, we excluded all focal areas where the potential foraging habitat was less than 20% (< 1125 m^2^) of the focal area. Therefore, focal areas with forest cover or urban covering more than 80% of the focal area were classified as unsuitable foraging areas [44, 58] and excluded from this part of the analyses. This procedure removed a total of 947 used locations and 123,551 available locations, resulting in a dataset of 5982 used versus 544,546 available locations (ratio: 1:91).

All numeric variables were scaled before being entered into the models. To obtain the posterior distribution, we directly simulated 1000 values from the joint posterior distribution of the model parameters using the “simulate_model” function of the package “parameters” [50]. The means of the simulated posterior distribution were used as estimates and the according 2.5% and 97.5% quantiles as upper and lower limits of the 95% credible interval (CrI). Effects were interpreted as meaningful when they differed from 0 (95% CrI not containing 0). To validate our models, we followed the method outlined by Johnson et al. [59] by regressing observed against predicted data (cross validation) (for details see Additional file 1: S2.1). All analyses were performed using the software R 4.1.0 [60].

## Results

### Vole- and small mammal indices

Both the vole and small mammal activity density showed fluctuations among sampling periods, regions, and years (see Additional file 1: S2.2 and S2.3 for detailed information). Small mammal activity density showed a similar pattern in crop-rotation and grasslands, with high values during the breeding period followed by a decline in autumn. Small mammal activity densities in forest and biodiversity structures also followed cyclic fluctuations but tended to reach their maximum later in the year, reaching into the non-breeding period. At the landscape level, this resulted in condensed small mammal availability during the non-breeding period, with high activity densities in and along forests and border structures and lower activity densities in adjacent agricultural areas (Fig. 1). For vole activity densities, grassland and border structures showed a pronounced difference between breeding and non-breeding periods: while both habitat categories showed low activity densities during the breeding period, they showed relatively high values during the non-breeding period (Fig. 1), resulting in a patchy distribution of vole availability at the landscape level during the non-breeding period compared to the breeding period.

### Home range size, location, and composition

Non-breeding home range size at the 95% isopleth of the minimum convex polygon ranged from 1.1 to 46.8 km^2^ (median: 6.8 ± 4.2 (MAD) km^2^, n = 51). Males had a median non-breeding home range of 4.6 ± 4.2 km^2^ (range: 1.1–46.8 km^2^, n = 27) and females of 8.3 ± 3.3 km^2^ (range: 4.0–25.3 km^2^, n = 24). Breeding home range size ranged from 1.1 to 38.2 km^2^ (median: 6.5 ± 4.9 km^2^, n = 46). Males had a median breeding home range of 5.7 ± 4.2 km^2^ (range: 1.1–12.8 km^2^, n = 24) and females of 9.3 ± 6.4 km^2^ (range: 2.4–38.2 km^2^, n = 22). The mean home range size did not differ between the breeding and the non-breeding period (0.10, CrI: − 0.13 to 0.38, n = 97), but males generally had smaller mean home ranges than females (− 0.49, CrI: − 0.87 to − 0.14). Within sex we could not detect any difference in home range size between breeding and non-breeding period (0.01, CrI: − 0.54 to 0.52). (Details see Additional file 1: S2.4).

Median distance from centroids of non-breeding home ranges to the previous breeding nest box was 1.1 ± 1.0 km (range: 0.1–18.1 km, n = 51). There was a difference in the distance from centroids of non-breeding home ranges to the previous breeding nest box where males had shorter distances than females (median males: 0.9 ± 0.6 km, range: 0.1–5.3 km, n = 27, median females; 1.6 ± 1.7 km, range: 0.2–18.1 km, n = 24; − 0.62, CrI: − 1.20 to − 0.07). Median distance from centroids of non-breeding home ranges to the future breeding nest box was 1.0 ± 1.0 km (range: 0.1–21.8 km, n = 42), with no difference between males and females (median males: 1.0 ± 0.8 km, range: 0.1–4.0 km, n = 23, median females 1.2 ± 1.0 km, range: 0.3–21.8 km, n = 19; − 0.63, CrI: − 1.27 to 0.00). In 83% (19 out of 23) of the cases, the males bred in the same nest box as the previous year and females in 37% (7 out of 19) of the cases. (Details see Additional file 1: S2.5).

During breeding the median distance from the nest box to the centroid of the home range was 0.5 ± 0.4 km (range: 0.1–3.9 km, n = 46). There were no sex differences in the distance between the breeding home range centroid and the breeding nest box (median males 0.5 ± 0.3 km, range: 0.2–2.0 km, n = 24, median female 0.6 ± 0.5 km, range: 0.1–3.9 km, n = 22; 0.00, CrI: − 0.53 to 0.60). (Details see Additional file 1: S2.5).

The median overlap of non-breeding and breeding home range was 38 ± 20% (range: 0–69%, n = 46). The median overlap of male home ranges was 41 ± 16% (range: 7–69% n = 24) and 33 ± 20% (range: 0–61%, n = 22) for females.

Non-breeding home ranges contained predominately crop rotation fields (43.5 ± 13.4%) followed by grasslands (16.1 ± 8.4%, intensive grassland, 5.6 ± 2.8% extensive grassland). Forest (13.4 ± 10%) and urban area (10.7 ± 8.3%) also covered a substantial amount of area, whereas border structures (1.6 ± 0.5%) and biodiversity structures (1.3 ± 0.7%) only covered a small amount of area (Fig. 2).

### Habitat selection

**Fig. 3:**
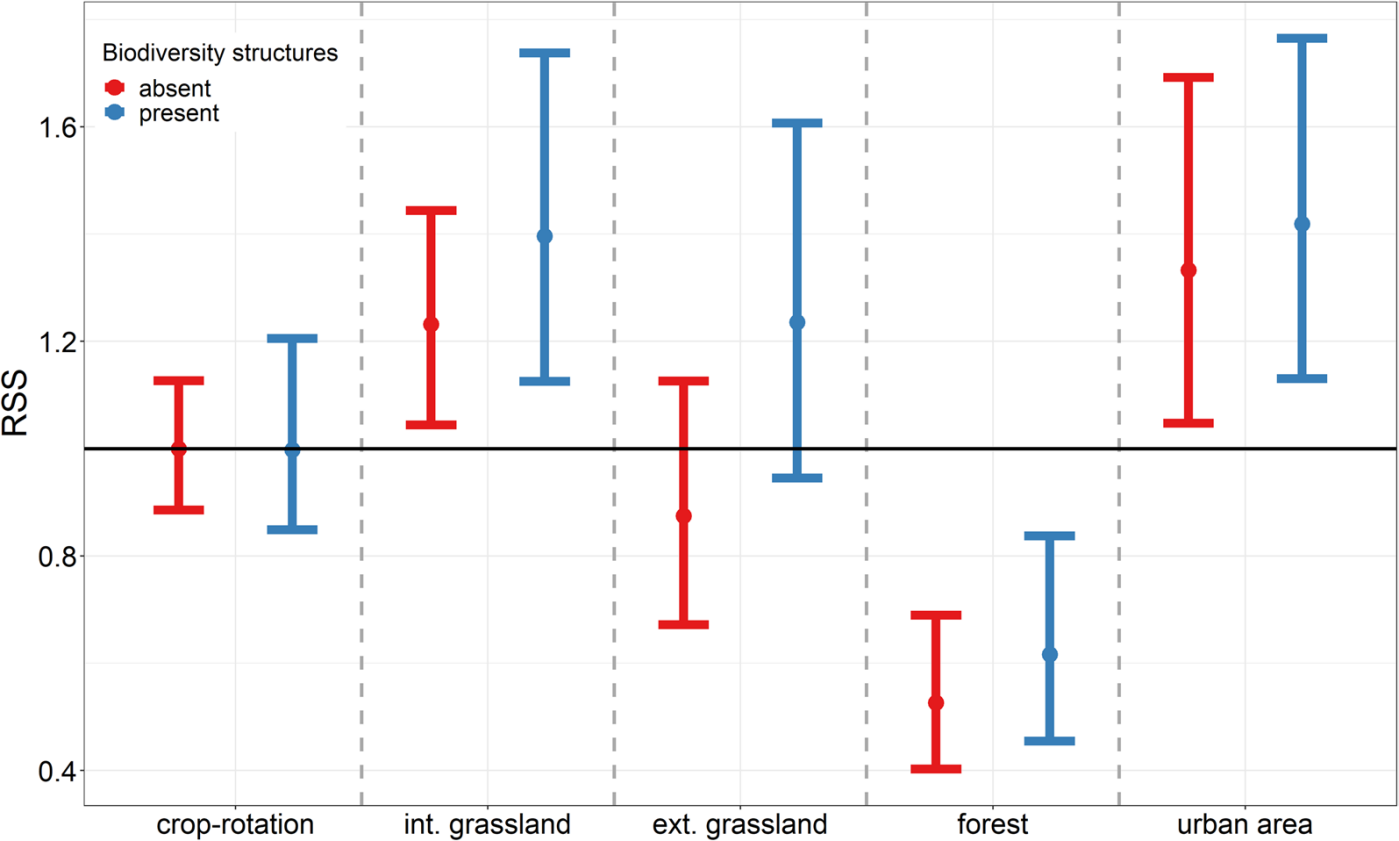
Mean estimates of RSS (exp(β)) and associated 95% credible interval of the posterior distribution of a logistic mixed effect model based on 65 individuals. Estimates are presented for biodiversity structures absent (red) and present (blue) for each habitat category. Habitat category and biodiversity structures were included as random slope and tag deployment as random intercept the reference category for the relative selection strength is represented by crop-rotation without biodiversity structures. Values shown for females

The resource selection function revealed that relative selection strength during the non-breeding period (RSS) (Table 1, Fig. 3) was highest for urban areas (RSS: 1.35 CrI: 1.09 to 1.63) followed by intensive grassland (RSS: 1.28 CrI: 1.07 to 1.55), extensive grassland (RSS: 1.07 CrI: 0.83 to 1.34) and was lowest for forest-dominated areas (RSS: 0.55 CrI: 0.41 to 0.72). RSS tells us the preference of the birds to choose any of the other categories over the reference category (crop rotation), given they are equally available and accessible. This means that the birds were 1.28 times more likely to choose areas dominated by intensive grassland than crop rotation if they were equally available. Further, the probability that a bird chose a location dominated by forest over one dominated by crop-rotation was 0.55 times lower. This gives the following, decreasing order of selection: urban area > intensive grassland > extensive grassland > crop rotation > forest. Both factors, structural diversity and the presence of biodiversity structures showed a positive estimate, but the effects were considered weak.

**Table 1:** Resource selection function for used and available focal areas within the non-breeding home ranges of barn owls

Fixed effects	Main effects β mean (95%CrI)	With interactions β mean (95%CrI)
Intercept	− 13.20 (− 13.31 to − 13.09)	− 13.18 (− 13.30 to − 13.07)
Sex m	**− 0.13 (− 0.22 to − 0.07)**	**− 0.13 (− 0.20 to − 0.06)**
Habitat category		
Intensive grassland	**0.25 (0.07 to 0.44)**	**0.21 (0.03 to 0.39)**
Extensive grassland	0.07 (− 0.18 to 0.29)	− 0.13 (− 0.40 to 0.17)
Forest	**− 0.60 (− 0.90 to − 0.33)**	**− 0.64 (− 0.92 to − 0.34)**
Urban area	**0.29 (0.08 to 0.48)**	**0.29** **(0.05** **to** **0.60**)
Biodiversity structures (present)	0.08 (− 0.10 to 0.28)	− 0.003 (− 0.19 to 0.20)
Structural richness	0.03 (− 0.05 to 0.11)	− 0.03 (− 0.20 to 0.11)
Intensive grassland × Biodiv. structures		0.13 (− 0.01 to 0.28)
Extensive grassland × Biodiv. structures		**0.35 (0.10 to 0.57)**
Forest × Biodiv. structures		0.16 (− 0.11 to 0.43)
Urban area × Biodiv. structures		0.07 (− 0.15 to 0.28)

Random effects	Variance (± sd)	Variance (± sd)
Deployment (intercept)	0.19 (± 0.44)	0.18 (± 0.43)
Intensive grassland (slope)	0.49 (± 0.70)	0.49 (± 0.70)
Extensive grassland (slope)	0.62 (± 0.79)	0.64 (± 0.80)
Forest (slope)	1.12 (± 1.06)	1.11 (± 1.05)
Urban area (slope)	0.61 (± 0.75)	0.63 (± 0.80)
Biodiv. structures 1 (slope)	0.57 (± 0.75)	0.56 (± 0.75)
Structural richness (slope)	0.10 (± 0.32)	0.10 (± 0.32)

We present the mean estimates of β and associated 95% credible interval of the posterior distribution of a logistic mixed effect model based on 65 individuals. Sex, habitat category, biodiversity structures and structural richness were introduced as explanatory variables, tag deployment as random intercept, and habitat category, biodiversity structures and structural richness as random slopes. The reference category is crop-rotation without biodiversity structures. First model without and second with interaction of habitat category with biodiversity structures

Effects whose credible interval does not include 0 are shown in bold

The presence of biodiversity structures had a positive effect on most habitat categories, but only the interaction with extensive grassland showed a meaningful effect (RSS: 1.42, CrI: 1.10 to 1.77) (Table 1, Fig. 3).

### Habitat selection, biodiversity structures, and prey

**Fig. 4:**
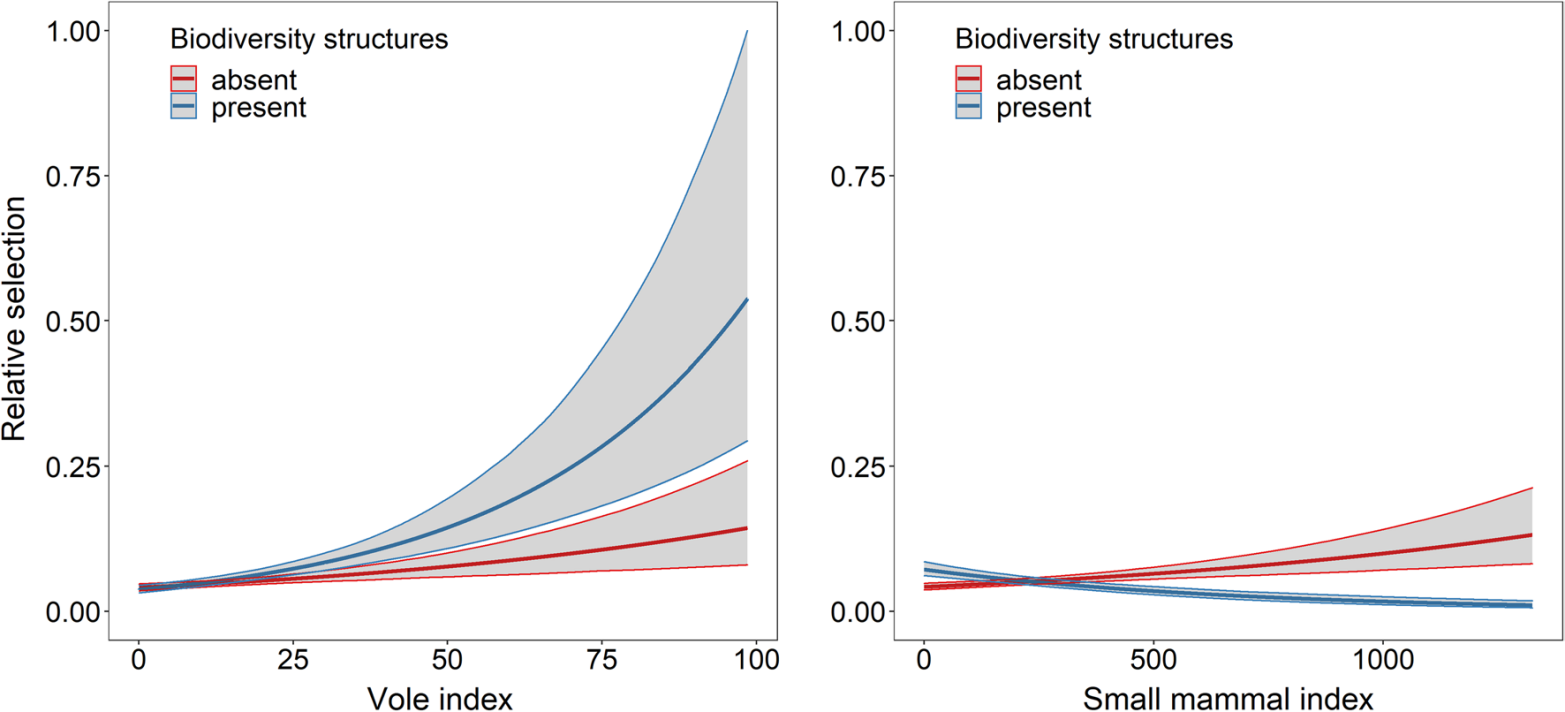
Relative selection strength for focal areas in relation to vole activity density per focal area (**A**) and small mammal activity density per focal area (**B**) with (blue) and without (red) biodiversity structures. We present mean estimates of β and associated 95% credible interval of the posterior distribution of the logistic mixed effect model of Table 2. Analyses are based on 74 deployments of 65 individuals. Upper limit of the 95% CrI of the highest vole activity density per focal area with biodiversity structures present was set to 1

To investigate the effect of biodiversity structures on the grassland preference found above, we calculated the vole and small mammal activity density per focal area and modelled the preference of owls as a function of vole and small mammal activity density within the focal area with biodiversity structures present or absent (Table 2, Fig. 4). The model estimates of the interaction of biodiversity structures and vole activity density per focal area suggested a positive selection when biodiversity structures were present (RSS: 1.23, CrI: 1.16 to 1.32, Fig. 4 A). The estimates of the interaction of biodiversity structures and small mammal activity density within focal area showed a weak negative effect if biodiversity structures were present (RSS: 0.68, CrI: 0.63 to 0.72, Fig. 4B). Generally, selection increased with increasing vole activity density in the focal area. For small mammal activity density within focal area, selection increased only in the absence of biodiversity structures. In fact, selection decreased slightly for areas with increasing small mammal activity density if biodiversity structures were present, however the effect size was small.

**Table 2:** Resource selection function for used and available focal areas within the non-breeding home ranges of barn owls

Fixed effects	β mean (95%CrI)
Intercept	− 13.07 (− 13.17 to − 12.96)
Sex m	**− 0.20 (− 0.30 to − 0.08)**
Small mammal activity density/focal area	**0.15** **(0.08** **to** **0.21)**
Vole activity density/focal area	**0.18** **(0.08** **to** **0.29)**
Biodiversity structures (present)	0.13 (− 0.04 to 0.30)
Small mammal activity density/focal area × Biodiv. structures	**− 0.39 (− 0.47 to − 0.32)**
Vole activity density/focal area × Biodiv. structures	**0.21 (0.15 to 0.28)**

Random effects	Variance (± SD)
Deployment (Intercept)	0.13 (± 0.39)
Small mammal activity density/focal area (slope)	0.04 (± 0.20)
Vole activity density/focal area (slope)	0.14 (± 0.38)
Biodiversity structures (slope)	0.48 (± 0.69)

We present the mean estimates of β and associated 95% credible interval of the posterior distribution of a logistic mixed effect model based on 65 individuals. Sex, vole activity density per focal area, small mammal activity density per focal area, and biodiversity structures were introduced as explanatory variables, tag deployment as random intercept, and vole activity density per focal area, small mammal activity density per focal area and biodiversity structures as random slopes. Vole and small mammal activity density per focal area was modelled in interaction with biodiversity structures

Effects whose credible interval does not include 0 are shown in bold

Focal areas with increased activity density within intensive grassland (RSS: 1.22, CrI: 1.08 to 1.36), extensive grassland (RSS: 1.09, CrI: 1.01 to 1.20) and border structures (RSS: 1.15, CrI: 1.02 to 1.35) were selected (Table 3), while focal areas with increased activity density within biodiversity structures were avoided. (RSS: 0.79, CrI: 0.71 to 0.88).

**Table 3:** Resource selection function for used and available focal areas within the non-breeding home ranges of barn owls

Fixed effects	β mean (95%CrI)
Intercept	− 13.05 (− 13.20 to − 12.91)
Sex m	**− 0.33 (− 0.53 to − 0.11)**
Total prey activity density intensive grassland/focal area	**0.20 (0.08 to 0.31)**
Total prey activity density extensive grassland/focal area	**0.09 (0.01 to 0.18)**
Total prey activity density biodiv. structures/focal area	**− 0.24 (− 0.34 to − 0.13)**
Total prey activity density crop rotation/focal area	0.03 (− 0.10 to 0.15)
Total prey activity density border structures/focal area	**0.15 (0.02 to 0.30)**
Structural richness	0.06 (− 0.05 to 0.17)

Random effects	Variance (± SD)
Deployment (intercept)	0.17 (± 0.41)
Total prey activity density intensive grassland/focal area (slope)	0.19 (± 0.44)
Total prey activity density extensive grassland/focal area (slope)	0.09 (± 0.30)
Total prey activity density biodiv. structures/focal area (slope)	0.11 (± 0.34)
Total prey activity density crop rotation/focal area (slope)	0.20 (± 0.45)
Total prey activity density border structures/focal area (slope)	0.38 (± 0.62)
Structural diversity	0.17 (± 0.41)

We present the mean estimates of β and associated 95% credible interval of the posterior distribution of a logistic mixed effect model based on 65 individuals. Sex, structural richness, and total prey activity density per habitat category and focal area were introduced as explanatory variables, tag deployment as random intercept, and total prey activity density per habitat category and focal area as random slopes

Effects whose credible interval does not include 0 are shown in bold

## Discussion

For a comprehensive picture of the ecology of a species, all phases of its life cycle should be studied. While many studies investigate habitat selection during the breeding period, fewer investigate the non-breeding period [1]. In this study we showed that small mammal distribution in different habitats varies over the annual cycle, with a general shift from annual crop-rotation to perennial habitats, which affects the availability of prey for predators and consequently the preference of their hunting grounds. Breeding and non-breeding home-range size were similar, but there was a small shift in the location of home-ranges which was more pronounced in females than males. The changes in prey activity density led to a mainly grassland-oriented habitat selection during the non-breeding period. Furthermore, our results showed the importance of biodiversity promotion areas and border structures within the intensively managed agricultural landscape.

### Home range size, location, and composition

With an average size of 6.8 km^2^ barn owl non-breeding home range size was similar to its breeding home range size and comparable to breeding home range sizes found in previous studies [44, 45, 58, 61]. Overall, females had larger home ranges than males in both periods. During breeding, home ranges of both sexes are closely bound to the breeding nest box, while the distance enlarged for females but not for males during non-breeding period. Overlap of breeding and non-breeding home range was higher for males than for females, indicating a shift in spatial placement of the home ranges during the non-breeding period in females. Such a pattern could emerge through a difference in nest site fidelity of the sexes during the non-breeding period. Previous studies have shown that in case of divorce (change of partner between years), males stay at the original nest site while almost all females change their nest site [62]. Our data showed a similar pattern, as females were more prone to change the nest site between breeding periods. Additionally, own, unpublished data suggests that males visit nest boxes more frequently and regularly than females outside the reproductive period. These findings together indicate that males exhibit higher nest site fidelity, resulting in regular visits to their nest box during the non-breeding period. That breeding site fidelity is more pronounced in males than in females has been shown for several migratory and non-migratory birds of prey species [63–65]. In contrast, little is known about site fidelity outside the breeding season. Our findings indicate that it may be advantageous for males to be present at the future breeding site early in the year. As a result of this behaviour, they may be more regionally bound and therefore less flexible to explore the surrounding areas during the non-breeding period. That site fidelity during the non-breeding season leads to less flexibility in space use but might lead to competitive advantage for breeding sites in spring has also been demonstrated for common kestrels [66]. Habitat composition of home-ranges is similar between the non-breeding (Fig. 2) and breeding period [44], with crop rotation covering the largest proportion of area. This is less surprising as farmers in our study area rarely change the area planted to a particular crop, but rather the field where they plant the crop.

### Habitat selection

Our data suggested the following decreasing order in habitat preference during non-breeding period: urban area > intensive grassland > extensive grassland > crop rotation > forest (Fig. 3). The reasons for such a strong selection towards urban area cannot be entirely answered in the frame of this study, but we formulate two hypotheses. First, owls roosting in buildings can save energy compared to roosting in trees especially in cold periods [67]. During the non-breeding period the pressure to save energy might be especially high, resulting in prolonged roosting behaviour during the night and thus select locations dominated by urban area. Even though baseline energy demand is higher during the non-breeding period, owls need less prey items, as they only need to feed themselves. As catching fewer prey might be less time-consuming, there would be more time available to roost. Second, microstructures around houses, such as gardens, hedges, or small orchards, might be suitable habitats of sufficient size for hunting during the non-breeding period. Being close to buildings and thus less exposed to weather conditions compared to hunting in open area might further favour this behaviour during periods of rough weather. However, as our data does not have the necessary resolution to allow to distinguish between roosting and active hunting behaviour, these two hypotheses remain untested.

The selection of intensive grassland (Fig. 3) is not very surprising, as previous studies showed that different types of grassland (pastures or meadows, intensively or extensively managed) are preferred by birds of prey during breeding [9, 44, 68, 69] and our analysis showed that for barn owls this preference seems to persist into the non-breeding period. Even though we see a positive selection of extensive grassland compared to crop rotation, the effect is very weak, which could be partly explained by the scarcity of these structures within the landscape (rareness effect). Further, the presence of biodiversity structures (hedges, trees, wildflower strips, rotational fallows) seemed to positively influence selection of grassland dominated areas (Fig. 3). This effect was pronounced in extensive grassland dominated areas and weaker in intensive grassland. However, the presence of biodiversity structures does not seem to influence the selection of the other habitat categories. Again, there are different explanations. While grasslands may provide suitable habitat for hunting, the neighbouring biodiversity structures could provide perching opportunities, allowing raptors to use a sit-and-wait hunting strategy instead of hunting on the wing [70]. Our own, but yet unpublished data suggest that hunting success in barn owls is higher if owls hunt from perches compared to hunting on the wings, possibly leading to a strong selection towards perch hunting in periods with high energetic baseline needs such as the colder non-breeding period [70–72]. Another possibility is that nearby biodiversity structures could cause a spill-over of small mammals into adjacent grasslands. Previous studies showed that semi natural habitats harbour high density of small mammals and can indeed act as a refuge and even as a source for reuse or recolonisation of intensively managed area in spring and summer [14, 15, 17, 18, 23–25, 27, 73].

Area dedicated to crop rotation, which represents the most abundant habitat category within the home ranges of our birds, showed a lower selection compared to intensive grasslands (Fig. 3). Crop rotations, especially cereal fields, are used by birds of prey during the breeding period [9, 44, 74, 75], however crop rotations fields look totally different in terms of vegetation coverage and food availability during the non-breeding period. In our analysis we did not find any preference for crop-rotations (cereal, colza, catch crop, maize, sugar-beet) over grassland during the non-breeding period. We also showed that small mammal activity density in crop-rotations drops drastically in late summer. This drop is most probably induced by harvesting which has been shown to affect small mammal populations either directly due to mortality caused by the harvesters, or due to a change in habitat suitability (reduced cover and food availability) for small mammals [14, 15, 18, 76]. While birds of prey benefit from the increased availability of prey in crop rotation fields during the breeding period, this effect may only persist until shortly after harvest when this habitat instantly become unattractive for small mammals and thus also for their predators.

### Habitat selection, biodiversity structures and prey

We showed that biodiversity structures (such as hedges, single trees, wildflower strips and rotational fallows) boost the preference for grassland dominated areas during the non-breeding period. This could be either due to an elevated abundance of perching opportunities when biodiversity structures are present, or due to high prey availability within these structures. We thus investigated whether habitat selection varies depending on prey activity density and the presence or absence of biodiversity structures (Fig. 4). We found a preference for areas with a high vole activity density within the focal area, which was even stronger when biodiversity structures were present. For small mammal activity density, the presence of biodiversity structures has a small opposite effect. Knowing that the two methods used in the small mammal monitoring represent a different spectrum of prey species, there are different possible explanations for the observed results.

The vole activity density represents almost exclusively the availability (presence, activity) of *Microtus* and *Arvicola* species (∼70% of barn owl prey, [36]). Selection for focal areas with a high vole activity density may be due to the altered accessibility of this prey type during the non-breeding period (Fig. 4). High, dense, and fast re-growing vegetation is thought to limit prey accessibility during the breeding period [9, 38, 68] but this might change later in the year. Indeed, the increase in vole signs observed from the breeding to the non-breeding period doesn’t solely represent an increase in vole abundance and activity but also partially reflects increased visibility of the signs for the observer during non-breeding period. While the signs, and therefore also voles themselves, are often hidden in long and dense vegetation during summer, they are more visible to the human eye and therefore also more accessible to the predator during the non-breeding period. Even though voles might be more accessible in the non-breeding than in the breeding period, hunting them during the non-breeding period could be quite time and energy consuming given that voles lead a rather secretive life and low temperatures and decreased vegetation cover further reduce vole activity [76, 77]. Nevertheless, hunting in areas with high vole activity density could be the most lucrative option during the non-breeding period, and perch hunting could be energetically beneficial. Grassland can provide high vole activity density, while the presence of biodiversity structures can provide the needed perching opportunities. Perching opportunities, especially small woody features, are crucial for the exploitation of agricultural landscapes by diurnal raptors [78] and their importance can even vary between periods. Studies on hunting behaviour of common kestrel have demonstrated a switch from use of hunting on the wing during summer, to perch hunting during winter [71, 72]. The observed habitat selection pattern may indicate that a similar, period specific, change in hunting behaviour also occurs in barn owls, but this remains to be tested.

Small mammal activity density represents the above-ground movements of a wide range of prey species, tending to include more mobile and active prey species such as the wood mice (home range: 4900 up to 14,400 m^2^, [18]). Consequently, the selection strength with regard to the activity density of small mammals also shows different patterns than with regard to the activity density of voles. While the presence of biodiversity structures seems beneficial at low small mammal activity density within focal areas, the positive effect of the structures vanishes towards higher activity densities (Fig. 4). The overall lower impact on focal area selection of small mammal activity density compared to vole activity density could result from the seasonal shift of prey availability. While small mammal activity density tended to be high among different habitat categories and especially in crop-rotation during the breeding period, this pattern changes during the non-breeding period. In agreement with previous studies on different small mammal species [14, 15, 17, 18] we observe a decrease inactivity density within crop plantations after harvest. Prey availability in crop-rotation dominated areas drops and activity only remains high in habitats which are unsuitable for hunting (forests).

Looking at the different foraging habitat categories separately, we see that focal areas with high total prey activity densities within grasslands and border structures get selected (Table 3). As we already know that owls prefer focal areas dominated by intensive grassland and areas with high vole activity density, the selection of focal areas with high prey activity density within intensive and extensive grassland is thus consistent with the previous findings. This further strengthens our conclusion that especially grasslands provide crucial food sources during the non-breeding period. The preference for focal areas with high total activity density in border structures is quite interesting as these structures usually only cover small areas within focal areas. That border structures are attractive habitat for voles [26] and consequently attract birds of prey has already been shown for little owls, common buzzards, common kestrels and black kites [38, 70]. The preference for border structures could be a combination of two effects: firstly, an increased availability of prey within border structures, and secondly, the fact that they are often found next to roads equipped with road sings and poles. These poles and road signs can serve as perches and could have a similar effect on selection as we hypothesized for the presence of biodiversity structures, by saving energy through perch hunting [70, 72]. The negative effect of high prey availability in biodiversity structures may seem contradictory at first. However not only prey abundance but also its accessibility is important for the selection of certain habitat structures [9, 38, 68]. Thus, a high total prey activity density per se does not necessarily favour selection, as these prey items are not necessarily accessible and can only be reached at the edge of these structures, or when they migrate into the adjacent grassland [38, 68].

### Conclusions

Our study highlights the need to investigate the full annual cycle to identify period-specific changes in animals’ behaviour ecology. During the non-breeding period owls showed a strong selection towards areas dominated by urban area, which are likely used for roosting. The patchier prey distribution during the non-breeding compared to the breeding period led to habitat selection towards grassland, which may mean that the ongoing conversion of permanent grasslands to crop rotation fields in Europe [79] could influence the demography of the species. While such a shift might only have moderate impacts on survival and reproduction during the breeding period, when prey is available within crop rotation, the decrease of grasslands could negatively affect survival during the non-breeding period due to shortage of prey. Biodiversity promotion areas, which have been shown to be an important driver of habitat selection during the breeding [44] and the non-breeding period, are mainly important as refuge and source habitat of small mammals [16, 23–25] but might be of limited suitability as direct hunting grounds for birds of prey [9, 38]. On the other hand, the woody structures within biodiversity promotion areas might serve as perching opportunities to facilitate hunting. Therefore, the distribution of such biodiversity promotion areas within the landscapes is of utmost importance for effective conservation measures. We therefore conclude that to effectively conserve birds preying on small mammals within the intensively managed landscape, we need to maintain and promote a diverse agricultural landscape interspersed with biodiversity promotion areas close to preferred hunting habitats. Future analyses should cover the effects breeding and non-breeding habitat composition and corresponding prey availability on survival and reproduction. Furthermore, it would be interesting to investigate whether conspecifics influence habitat selection. The density of animals using a particular habitat can influence the habitat selection decision of other individuals [80]. Given the large fluctuations that barn owl populations can undergo in temperate regions, the influence of density on habitat selection could vary greatly from year to year and over the course of a year.

## Supplementary Information

**Additional file1**: S1.1: Small mammal sampling regions. S1.2: Small mammal sampling design visualization. S1.3: Information extraction from the federal layer TLM3D. S1.4: Information extraction from cantonal layers on agricultural fields. S1.5: Merged habitat types for habitat categories. S2.1: Model validations. S2.2: Small mammal activity density. S2.3: Vole activity density. S2.4: Models home range. S2.5: Model distance centroid nest box.

## Data Availability

Datasets and the R script to reproduce the resource selection functions, as well as their validations, are available under: 10.5281/zenodo.7664853. The GPS data are stored in Movebank (www.movebank.org), under the project named “Barn owl Winter” (ID 1433219445) and are available on reasonable request. The habitats maps produced during the study are available from the corresponding author on reasonable request.
