# Correction: Influence of prey availability on habitat selection during the non-breeding period in a resident bird of prey

**DOI:** 10.1186/s40462-023-00391-4

**Published:** 2023-05-09

**Authors:** Roman Bühler, Kim Schalcher, Robin Séchaud, Stephanie Michler, Nadine Apolloni, Alexandre Roulin, Bettina Almasi

**Affiliations:** 1 Swiss Ornithological Institute, Seerose 1, Sempach, 6204 Switzerland; 2 Department of Ecology and Evolution, University of Lausanne, Building Biophore, Lausanne, 1015 Switzerland; 3 Agroecology and Environment, Agroscope, Reckenholzstrasse 191, Zurich, 8046 Switzerland

**Correction to**: Movement Ecology (2023) 11:14

10.1186/s40462-023-00376-3

Following publication of the original article [1], it was noted that due to a typesetting mistake, the incorrect file for Additional file 1 was processed.

The correct file for Additional file 1 is attached to this Correction and has been corrected in the original article. The publisher apologises to the authors and readers for the inconvenience caused by this error.

## Electronic supplementary material

Below is the link to the electronic supplementary material.

**Additional file 1**: S1.1: Small mammal sampling regions. S1.2: Small mammal sampling design visualization. S1.3: Information extraction from the federal layer TLM3D. S1.4: Information extraction from cantonal layers on agricultural fields. S1.5: Merged habitat types for habitat categories. S2.1: Model validations. S2.2: Small mammal activity density. S2.3: Vole activity density. S2.4: Models home range. S2.5: Model distance centroid nest box.
