# Supplementary material for: Influence of prey availability on habitat selection during the non-breeding period in a resident bird of prey
Source: Mov Ecol. 2023 Mar 7;11:14. doi: 10.1186/s40462-023-00376-3 (PMC9990330; doi:10.1186/s40462-023-00376-3)
Supplement: Supplementary file 1 — Additional file1: S1.1: Small mammal sampling regions. S1.2: Small mammal sampling design visualization. S1.3: Information extraction from the federal layer TLM3D. S1.4: Information extraction from cantonal layers on agricultural fields. S1.5: Merged habitat types for habitat categories. S2.1: Model validations. S2.2: Small mammal activity density. S2.3: Vole activity density. S2.4: Models home range. S2.5: Model distance centroid nest box. [file 40462_2023_376_MOESM1_ESM.pdf]

Supplementary material:

S1.1 Small mammal sampling regions:

Each of the four regions contsist of 4 plots: red: plain of Orbe, yellow: plain of the Broye, blue: Haut-Fribourg and green: Gros de Vaud region. Nest box locations are indicated by points.

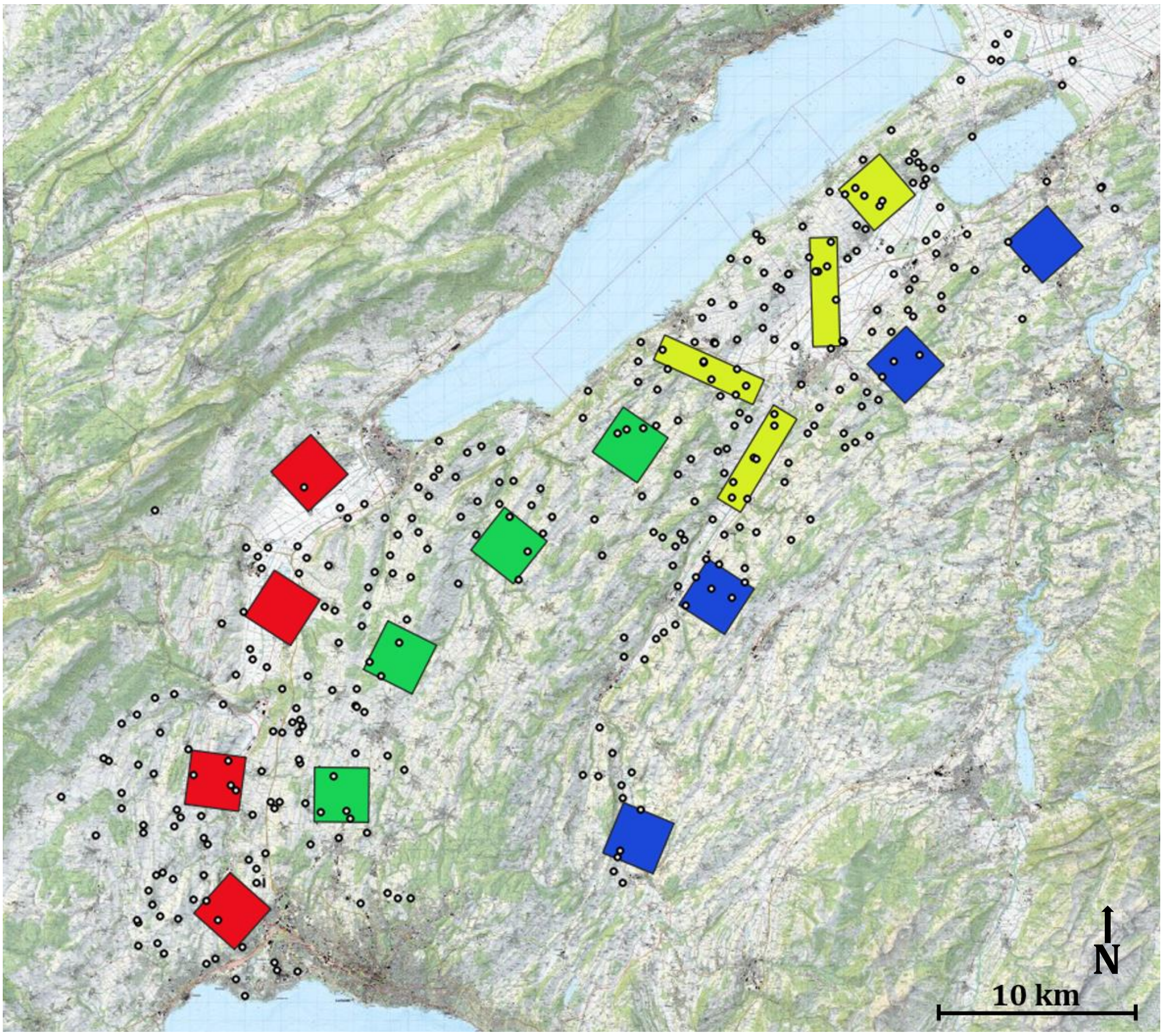

S1.2 Small mammal sampling design visualization

Each of the 4 habitat types was sampled with 9 transects/trackplates within each of the 4 plots of one region (upper left and right). This results in 36 transects/trackplates per habitat type per region per 2-month session (upper right). Within the 9 km^2 plots the transects and trackplates were laid that they covered the whole area over the duration of the year which consisted out of 6 sessions (lower left). Over the duration of one year each habitat within each region was sampled by 144 transects/trackplates (lower right).

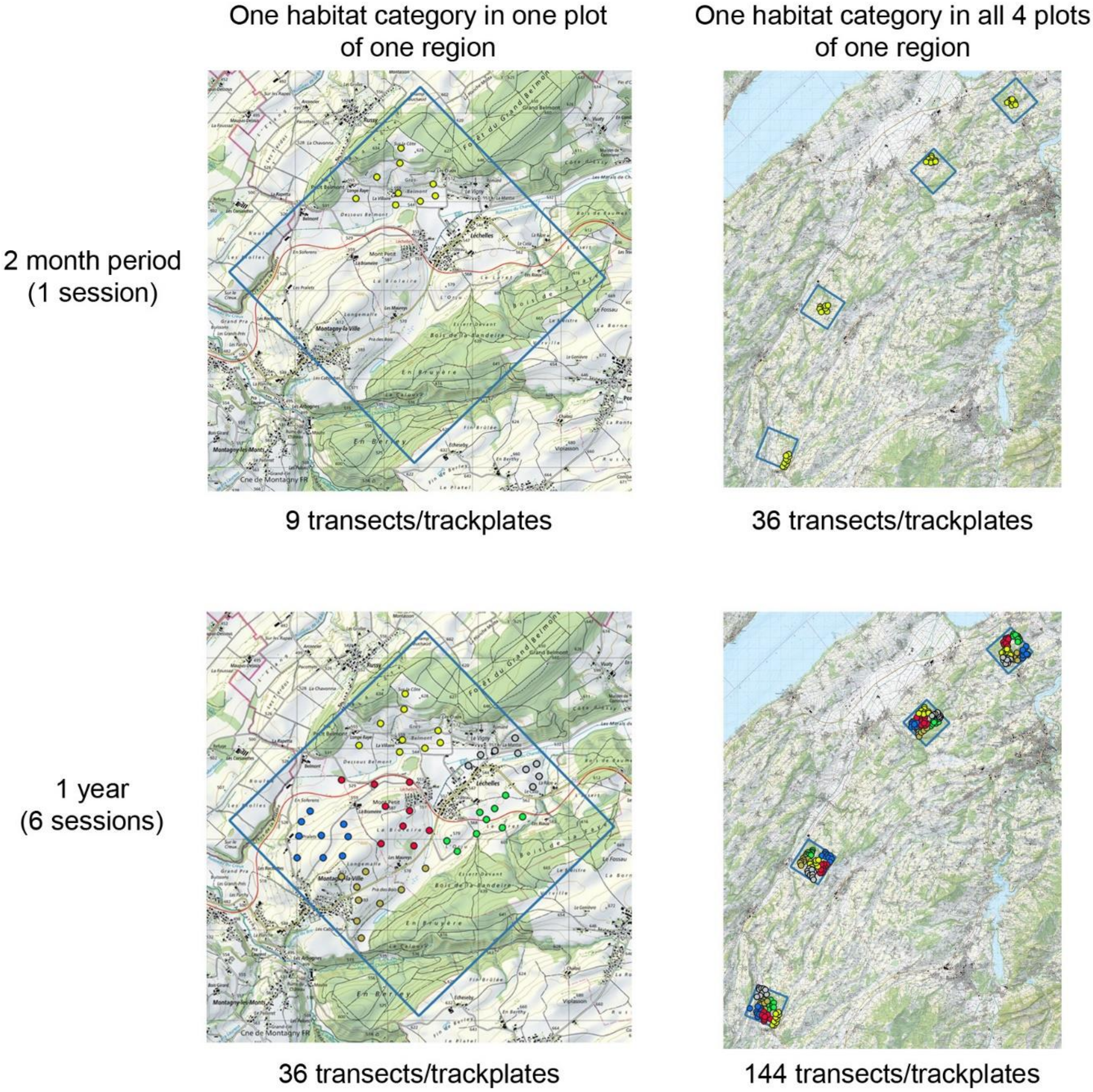

S1.3 Information extraction from the federal layer TLM3D

Below a table with the extracted categories and their treatment:

TLM 3D category	Buffer	Further treatment
buildings	+ 10 m (mean width)	holes < 500m^2 closed
highway	+ 20 m (mean width)	
motorway	+ 12 m (mean width)	
railway	+ 5 m (mean width)	
10 m street	+ 12 m (mean width)	
8 m street	+ 9.2 m (mean width)	
6 m street	+ 7.2 m (mean width)	
4 m street	+ 5.2 m (mean width)	
2 m path	+ 2.3 m (mean width)	
1 m path	+ 1.5 m (mean width)	
hedges	+ 1 m	
forest	- 3 m	holes < 500m^2 closed
forest edge	+ 7 m around forest	
border structures	+1 m around all streets/railway	

S1.4 Information extraction from cantonal layers on agricultural fields

For all locations in canton Vaud, agricultural land use was available as a GIS-layer from the cantonal authority. For canton Fribourg a layer with information about permanent cultures only was available and we therefore mapped the agricultural land use within an area of 1.5 km (which corresponds to the mean home range used by breeding barn owls (Almasi et al. 2013) around breeding sites of equipped owls during winter. After the GPS modules were recovered, areas of the home ranges which laid outside the already mapped 1.5 km radius were mapped as soon as possible. The information from the cantonal layers was extracted and merged with the data from the habitat mapping. To be able to merge the cantonal layer with the mapped layer we had to bring them to the same resolution and defined the following categories: Intensive grassland, extensive grassland, artificial grassland, intensive pasture, extensive pasture, winter cereal, colza, catch crop, wildflower strips and rotational fallows. As cantonal layers hold information about the culture present during summer of the respective year, we needed to combine the information from two years, according to the sowing time of the culture.

Finally, the different layers from the different sources have been clipped with each other to eliminate overlaying polygons. To fasten the workflow and to be able to efficiently extract the habitat information later in the analysis, the polygon layers have been rasterized on a resolution of 25x25m sized cells. This resolution was chosen according to the accuracy of our tags (mean accuracy: 20 m) and the lowest resolution of our base raster which was 2.5 m.

S1.5 Merged habitat types for habitat categories

Categories were merged according to their similar appearance in the field during the non-breeding period (agricultural fields) or because of strong correlations. Before merging we made sure that the habitat types showed similar selection directions in the raw data (ratio available vs. used)

Habitat category merged	Habitat type	Nr. of crop type (cantonal layers)
intensive grassland	intensive pasture<br>intensive meadow<br>artificial grassland	601/602/613/621/632/698/702/703/704<br>720/616
extensive grassland	extensive pasture<br>extensive meadow	611/612/634/697/851/852/857/858/622<br>617/618/625/693/694/930
biodiversity structures	rotational fallows<br>wildflower strips<br>hedges<br>single trees	555/556/557/559/572/904/905/908/998
crop-rotation	winter cereals<br>colza<br>catch crop<br>maize<br>potato<br>sugar beet<br>other	501/502/504/505/506/507/508/509/511<br>512/513/514/515/516/519/526/527/521<br>522/523/524/525/528/531/534/535/536<br>537/538/539/541/542/543/544/545/546<br>547/548/549/551/553/554/566/567/568<br>569/573/590/591/574/592/594/595/597<br>598/631/701/705/706/707/708/709/710<br>711/712/713/714/715/717/718/719/721<br>722/725/731/735/797/798/801/803/806<br>807/808/847/848/897/898/909
forest	forest<br>forest edge	from TLM3D
urban area	buildings<br>paths/streets/roads/<br>railways with buffer<br>1m	from TLM3D

S2.1 Model validations

We separated the dataset into training and test data, by randomly choosing 3 males and 3 females per season (12 individuals test dataset, 58 individuals training dataset). We then run an RSF using the training dataset, extracting the values for relative probability of selection. These values were separated into deciles of about equal size and the expected number of observations was calculated in each bin. To visually inspect the relationship, we plotted the observed number of used locations relative to the expected number in each bin and extracted the slope and intercept of the linear regression laid trough the locations. We repeated the process 10 times and calculated mean slope, intercept and spearman correlation among the 10 trials as measure for model performance.

Model	Spearman rank corr.	Intercept	Slope	R2
Model dominant habitat category	0.78 (±0.11)	0.03 (±0.02)	0.71 (±0.22)	0.62 (±0.20)
Model dominant habitat category interaction	0.86 (±0.10)	0.03 (±0.02)	0.66 (±0.18)	0.70 (±0.19)
Model total prey activity density	0.80 (±0.11)	0.02 (±0.01)	0.78 (±0.15)	0.72 (±0.23)
Model prey activity density per habitat category	0.71(±0.15)	0.02 (±0.03)	0.76 (±0.29)	0.41 (±0.26)

S2.2 Small mammal activity density

Numbers of small mammal traces for track-plates (small-mammal index) were analysed by fitting a generalized additive model. While the total number of traces, on the plates served as response variable, region, observation round, habitat category and temperature were included as explanatory variables. Fitted values for a given time for each habitat structure in each region (red: plain of the Broye, blue: Haut-Fribourg, green: Gros de Vaud, black: plain of Orbe) are estimated and used as an index representing small mammal activity density.

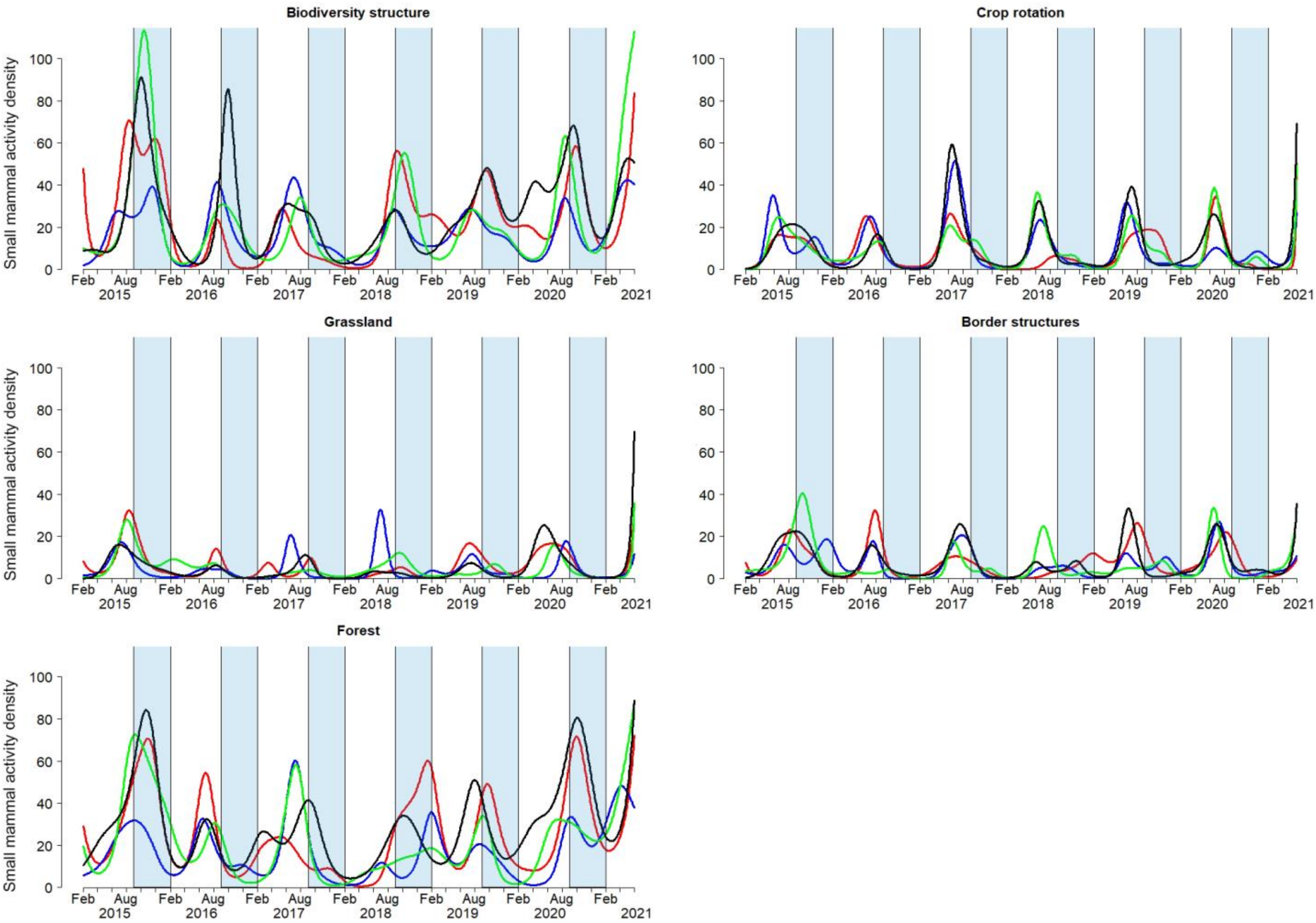

S2.3 Vole activity density

Numbers of vole traces for transects (vole index) were analysed by fitting a generalized additive model. While the total number of traces per transect served as response variable, region, observation round, habitat category and temperature were included as explanatory variables. Fitted values for a given time for each habitat structure in each region (red: plain of the Broye, blue: Haut-Fribourg, green: Gros de Vaud, black: plain of Orbe) are estimated and used as an index representing vole activity density.

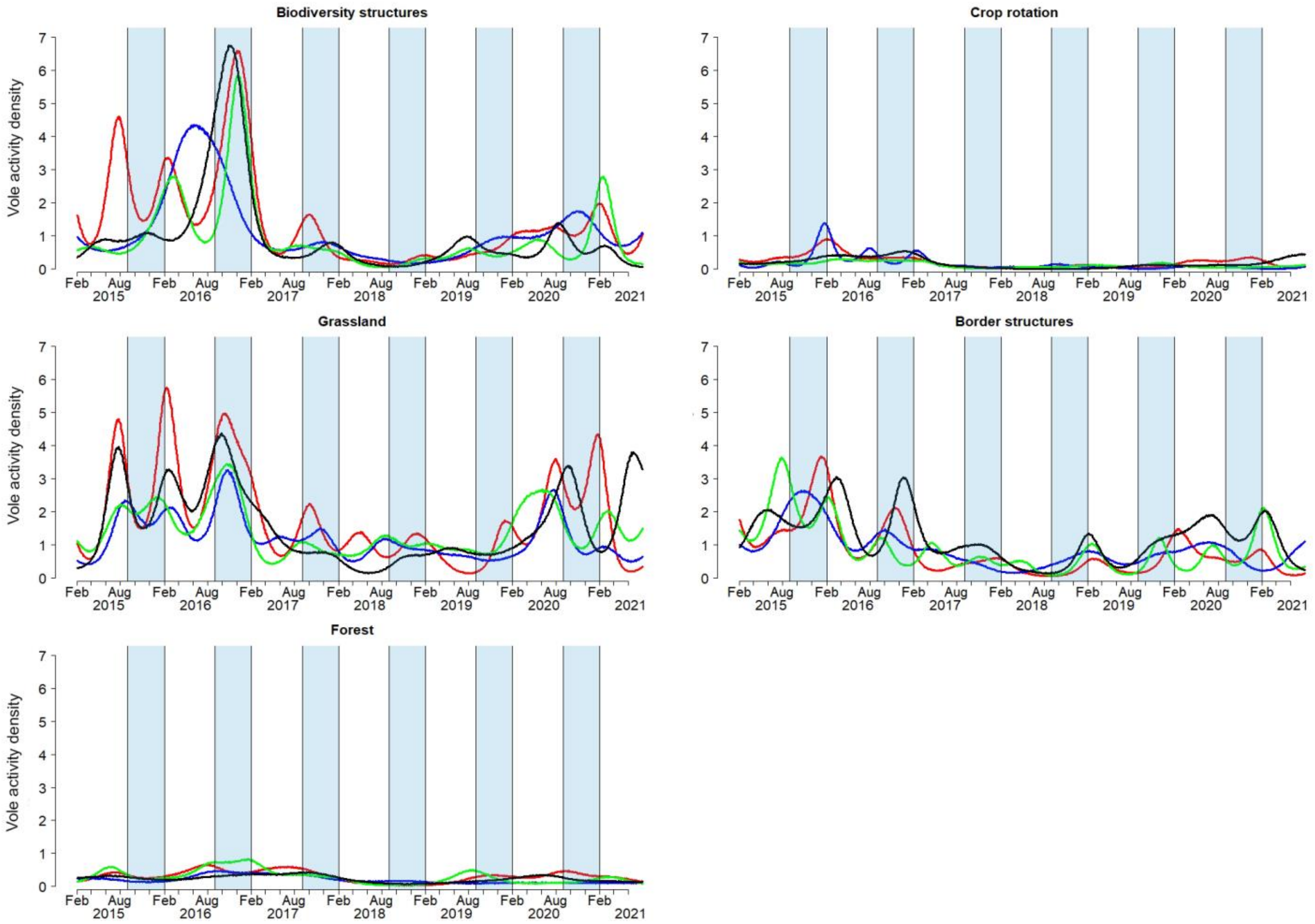

S2.4 Models home range

Linear models with log-transformed home range size as response variable and season (breeding/non-breeding), sex and year as explanatory variables. Individual identity was included as random effect. We present the mean estimates of ß and associated 95% credible interval of the posterior distribution based on 51 individuals (27 males, 24 females).

Fixed effects	Main effects ß mean (95%CrI)	With interaction ß mean (95%CrI)
Intercept [log]	2.22 (1.89–2.54)	2.20 (1.86–2.59)
Sex m	**-0.49 (-0.87– -0.14)**	**-0.47 (-0.94– -0.01)**
Season non-breeding	0.10 (-0.13–0.38)	0.11 (-0.24–0.52)
Year	-0.28 (-0.59–0.01)	-0.28 (-0.59–0.05)
Season non-breeding: sex m		0.01 (-0.54–0.52)
Random effects	Variance (±SD)	Variance (±SD)
Individual (Intercept)	0.20 (±0.44)	0.19(±0.44)

S2.5 Model distance centroid to nest box

We measured: 1) the distance of the centroid of the breeding home range to the breeding nest box (N=46, 24 males, 22 females), 2) the distance of the centroid of the non-breeding home range to the previous breeding nest box (N=51, 27 males, 24 females), 3) the distance of the centroid of the non-breeding home range to the future breeding nest box (N=42, 23 males, 19 females). Linear model with log-transformed distance as response and the type of distance (1,2,3) as well as sex as explanatory variable. Individual and nest box id were included as random effects. We present the mean estimates of ß and associated 95% credible interval of the posterior distribution.

Fixed effects	ß mean (95%CrI)
Intercept [log]	6.36 (5.99–6.81)
Dist. non-breeding -previous nest box	**1.04 (0.63–1.42)**
Dist. non-breeding -future nest box	**1.07 (0.57-1.55)**
Sex m	0.00 (-0.53-0.60)
Dist. non-breeding-previous nest box: sex m	**-0.62 (1.20- -0.07)**
Dist. non-breeding-future nest box: sex m	-0.63 (-1.27-0.00)
**Random effects**	**Variance (±SD)**
Individual (Intercept)	0.12 (±0.34)
Breeding site: Individual (Intercept)	0.34 (±0.58)
